# TOWARDS Study: Patient-Derived Xenograft Engraftment Predicts Poor Survival in Patients With Newly Diagnosed Triple-Negative Breast Cancer

**DOI:** 10.1200/PO.23.00724

**Published:** 2024-07-29

**Authors:** Christos Vaklavas, Cindy B. Matsen, Zhengtao Chu, Kenneth M. Boucher, Sandra D. Scherer, Satya Pathi, Anna Beck, Kirstyn E. Brownson, Saundra S. Buys, Namita Chittoria, Elyse D'Astous, H. Evin Gulbahce, N. Lynn Henry, Stephen Kimani, Jane Porretta, Regina Rosenthal, John Ward, Mei Wei, Bryan E. Welm, Alana L. Welm

**Affiliations:** ^1^ Department of Internal Medicine, Division of Oncology, Huntsman Cancer Institute, University of Utah, Salt Lake City, UT; ^2^ Department of Surgery, Huntsman Cancer Institute, University of Utah, Salt Lake City, UT; ^3^ Department of Oncological Sciences, Huntsman Cancer Institute, University of Utah, Salt Lake City, UT; ^4^ Department of Internal Medicine, Division of Epidemiology, Salt Lake City, UT; ^5^ Huntsman Cancer Institute Clinical Trials Office, Salt Lake City, UT; ^6^ Department of Pathology, Huntsman Cancer Institute, University of Utah, Salt Lake City, UT; ^7^ Current Address: Department of Internal Medicine, University of Michigan, Ann Arbor, MI

## Abstract

**PURPOSE:**

Assessing risk of recurrence for nonmetastatic triple-negative breast cancer (TNBC) is a key determinant of therapeutic strategy. The best predictor of recurrence risk is failure to achieve a pathologic complete response after preoperative chemotherapy, but it imperfectly correlates with the definitive end points of relapse-free and overall survival (OS). The inability to accurately predict recurrence has led to increasingly toxic treatment regimens for patients with early-stage TNBC. Better assays for recurrence risk are needed to tailor aggressive therapy for patients who need it and avoid overtreatment and unnecessary toxicity for those at low risk. The purpose of this study was to determine if patient-derived xenograft (PDX) engraftment of newly diagnosed breast tumors can serve as an accurate predictor of recurrence and death from breast cancer.

**METHODS:**

This study was a blinded noninterventional trial comprising 80 patients with newly diagnosed, nonmetastatic, estrogen receptor (ER)-negative or ER-low breast cancer.

**RESULTS:**

PDX engraftment was strongly associated with relapse in 1 year: 8 of 18 (44.4%) patients whose tumors engrafted relapsed versus 1 of 62 (1.6%) patients whose tumors did not engraft (*P* < .0001). Patients whose tumors engrafted had a hazard ratio (HR) for relapse of 17.5. HRs for OS and breast cancer-specific survival in PDX+ patients were 21.1 and 39.5, respectively.

**CONCLUSION:**

We report that the ability of a tumor to engraft as a PDX predicts early recurrence by serving as a functional readout of aggressiveness and prospectively identifies the most devastating tumors. This provides new opportunity to develop surrogate assays, such as biomarkers of engraftment, which will extend the clinical feasibility of this finding.

## Teaser

PDX engraftment predicts early relapse and death from TNBC more accurately than standard assessments.

## INTRODUCTION

In nonmetastatic triple-negative breast cancer (TNBC), the achievement of pathologic complete response (pCR) with preoperative therapy is associated with better long-term outcomes,^1,2^ and pCR serves as an intermediate end point to measure the efficacy of preoperative therapy.^3^ However, improvements in pCR rate achieved with more intensive preoperative regimens have not consistently been associated with meaningful improvements in event-free and overall survival (OS).^1,4-7^ Clinical trials with adjuvant chemotherapies to mitigate risk of recurrence in patients with residual TNBC have not been uniformly successful.^8-12^ Better biomarkers are needed to identify patients at high risk for recurrence, so more effective treatment regimens can be developed for those with a true high risk of recurrence.

**CONTEXT**

**Key Objective**
To determine if the ability of a newly diagnosed breast tumor to grow as a patient-derived xenograft (PDX) serves as a prognostic predictor of patient outcome.
**Knowledge Generated**
In a blinded noninterventional trial with 80 patients, we found that PDX engraftment is a strong, independent prognostic factor for early relapse and death from breast cancer in triple-negative breast cancer and hormone receptor-low, human epidermal growth factor receptor 2–negative patients and is more accurate and specific than standard assessments for recurrence risk.
**Relevance**
Tumor engraftment assays measure the aggressiveness of tumors, identifying patients who are at highest risk for poor outcome and may benefit from additional treatment. Future directions are to validate the findings with longer follow-up time, and to develop a clinically feasible surrogate biomarker for engraftment as a future prognostic test.

High rates of attrition for new oncology drugs^13^ have motivated development of patient-derived xenografts (PDXs) for preclinical studies.^14-21^ PDXs retain most of the phenotypic and molecular features of the originating tumors,^15,17,22^ allowing for more accurate predictions about the activity of novel drugs in the clinic.^23^ A limitation of PDX models is that not all tumors successfully engraft, with variable take rates depending on breast cancer subtype.^21^ We and others have noted correlations between clinicopathologic features of tumors and ability to form PDX, where engraftment correlates with a more aggressive phenotype.^24-33^ In a small retrospective study, we reported that the ability of primary breast tumors to engraft as PDX was predictive of OS.^15^ These observations motivated the TOWARDS clinical trial (Towards Personalized Medicine—Patient-Derived Breast Tumor Grafts as Predictors of Relapse and Response to Therapy, a Double Blinded Study), in which we tested whether PDX engraftment could prospectively predict disease recurrence in patients with newly diagnosed estrogen receptor (ER)-low or ER-negative breast cancer. The primary objective of the study was to prospectively evaluate the relationship between patient outcome and the ability of a primary tumor, collected before neoadjuvant therapy, to engraft as a PDX. The secondary and primary clinical end points, respectively, were disease-free survival 1 and 3 years after chemotherapy and surgery with curative intent. Here, we report the 1-year end point, where we found a highly significant association between PDX engraftment and poor outcome that was more accurate and more specific than pCR for predicting early (1-year) recurrence.

## METHODS

### Institutional Review Board Approval

The study was approved by the University of Utah Institutional Review Board (IRB; #00091596) and the Department of Defense (DOD) Human Research Protection Office (HRPO; #A-18507) and conducted in accordance with the Declaration of Helsinki. As a diagnostic, observational trial, it was not registered on ClinicalTrials.gov; the results are being publicly reported as required by the funding source. Informed consent was obtained from all patients before initiating study procedures, and consent was updated when deemed necessary by the IRB for protocol amendments. The full IRB- and HRPO-approved protocol is included in supplemental information.

### Patient Eligibility

TOWARDS was a noninterventional trial with 80 patients. Patients and investigators were blinded to engraftment results and outcomes. Eligible patients had newly diagnosed invasive breast cancers that were previously untreated, nonmetastatic, ER negative or low (≤10%), human epidermal growth factor receptor 2 (HER2)^34,35^ any status, and a primary tumor or lymph node lesion ≥1.5 cm. Patients had to receive preoperative chemotherapy at physician's discretion per standard of care (SOC). Clinical stage at diagnosis was determined by standard tumor, node, metastasis classification. Tumor tissue was acquired via 14-16 g core needle biopsy before initiation of preoperative chemotherapy. Most tumor specimens were acquired concurrent with implantable venous port placement.

During the course of the study, it became apparent that information generated from the PDX models could have clinical implications.^17^ The protocol was amended and approved by the IRB and DOD to make provisions for unblinding the treating physician to share potentially important information generated from the models. All participating physicians were trained about the study and completed the Statement of Investigator Form (US Food and Drug Administration 1572). The fact that the results were not obtained in a Clinical Laboratory Improvement Amendments setting was disclosed, and the physician could decide whether to use the study information.

### Definition of End Points

pCR was defined as the absence of invasive carcinoma in the surgical pathology of the primary tumor bed and excised lymph nodes. Relapse-free interval (RFI) was defined as the interval between definitive surgery and histologic confirmation of local, locoregional, or systemic recurrence or death from breast cancer.^36^ OS was defined as the interval between definitive surgery and death of any cause.^36^ Breast cancer-specific survival (BCSS) was defined as the interval between definitive surgery and death from breast cancer. Adverse events were reported according to the National Cancer Institute Common Terminology Criteria for Adverse Events version 4.

### Tumor Engraftment

Tumors were surgically engrafted into 3-4 week old female non-obese diabetic/severe combined immunodeficiency (NOD/SCID) mice (Jax#001303) as previously described.^15,17,37^ No Matrigel or hormone supplements were used. PDX were considered established after they were serially passaged in mice for at least two generations, sufficient tumor was cryopreserved, and they were validated by immunohistochemistry.^17^

### Statistical Analyses

#### Analyses Prespecified in the Approved IRB Protocol

The primary end point (3-year disease-free survival) has not yet been reached; here, we report on two prespecified secondary objectives/end points: association of PDX engraftment with 1-year disease-free survival in the overall population and relationship between PDX engraftment and pathological response to neoadjuvant chemotherapy. All patients were followed for at least 1 year. Kaplan-Meier curves and log-rank tests were used for event analysis stratified by pCR status and PDX engraftment.

#### Post Hoc Analyses

The Fisher exact test was used to determine the association between pCR or engraftment and recurrence in 1 year. We analyzed 1-year outcomes in the ER-low HER2-negative and TNBC subgroups. We also tested accuracy (sensitivity and specificity) of PDX engraftment and pCR in predicting 1-year recurrence with Wilcoxon signed rank tests. To identify predictors of an end point event, Cox models and likelihood ratio tests were used.

## RESULTS

### Patient Characteristics

**FIG 1.**
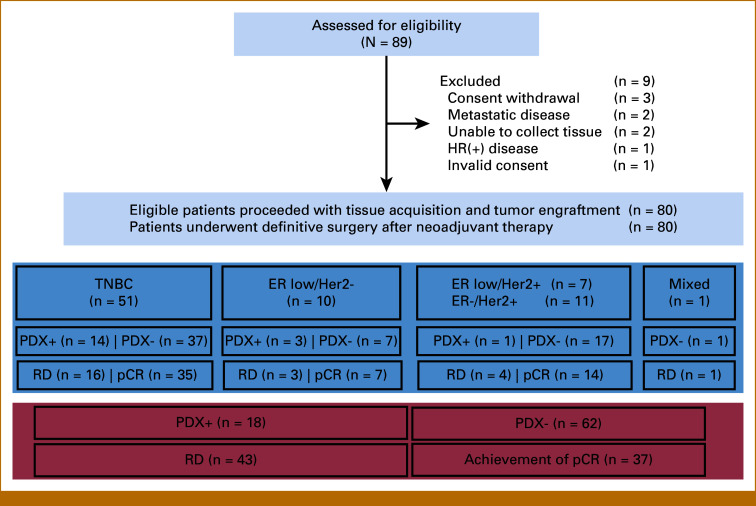
Flow diagram for the TOWARDS study. ER, estrogen receptor; HER2, human epidermal growth factor receptor 2; pCR, pathologic complete response; PDX, patient-derived xenograft; PDX+, successful engraftment; PDX–, no engraftment; RD, residual disease; TNBC, triple-negative breast cancer.

Eighty-nine patients were assessed for eligibility. Eighty patients were eligible and were enrolled, whereby tumor tissue was acquired before preoperative chemotherapy and implanted into mice (Fig 1). Patient characteristics are outlined in Table 1. All patients received SOC preoperative regimens, and the trial required an Eastern Cooperative Oncology Group performance status of 2 or better. Some patients had preoperative treatment dose reduced, delayed by >1 week, or prematurely terminated, primarily due to adverse events. In two patients with metaplastic TNBC, preoperative chemotherapy was prematurely changed and/or terminated because of interval disease progression. There were 39 patients (49%) with TNBC or ER-low, HER2-negative breast cancer who had residual disease after preoperative chemotherapy. Eighteen of them (46%) received capecitabine in the adjuvant setting.

**TABLE 1.** Patient Demographics and Tumor Characteristics (all patients)

Patient Characteristics (n = 80)	Value
Age, median (range)	52.9 (28.6-79.1)
Race/ethnicity,^a^ No.	
White	65
Hispanic/Latino	6
Asian/Filipino	3
Black	1
Native American	1
Other/undisclosed	4

Tumor Characteristic	Value
Clinical stage, No.	
IA	3
IIA	43
IIB	20
IIIA	6
IIIB	3
IIIC	5
Nodal status at diagnosis, No.	
N0	51
N1	22
N2	2
N3	5
Size of primary tumor, cm	
Median	3.4
Mean	3.7
Range	1.9-8.9
Histologic type, No.	
Invasive ductal carcinoma	65
Invasive ductal carcinoma with special features^b^	13
Metaplastic carcinoma	2
Histologic grade, No.	
Grade 2	13
Grade 2-3	2
Grade 3	65
Receptor status, No.	
TNBC	51
ER and/or PR 1%-10%, HER2-negative	10
ER and PR negative, HER2-positive	11
ER and/or PR 1%-10%, HER2-positive	7
Mixed (TNBC and HR-negative/HER2-positive)	1
Preoperative chemotherapy (HER2–; n = 62), No. (%)	
Dose dense AC-T^c^	52
Dose dense AC-paclitaxel carboplatin	4
Docetaxel cyclophosphamide^d^	4
Weekly carboplatin—paclitaxel	2
Adjuvant capecitabine in residual disease (n = 39)	18 (46)
Assigned regimen completed	26 (42)
Assigned regimen incomplete^e^	36 (58)
Germline testing in HER2– (performed in 43/62 patients), No.	
Negative and variants of unknown significance	30
Pathogenic or likely pathogenic mutations^f^	13
Preoperative chemotherapy (HER2+; n = 18), No. (%)	
TCHP^g^	14 (78)
TCH^h^	1 (5)
Dose dense AC followed by THP^i^	3 (17)
Adjuvant trastuzumab emtansine in residual disease (n = 4)	2 (50)
Assigned regimen completed	8 (44)
Assigned regimen incomplete^e^	10 (56)

Abbreviations: AC, doxorubicin + cyclophosphamide; CMF, cyclophosphamide-methotrexate-5-fluorouracil; ER, estrogen receptor; HER2, human epidermal growth factor receptor 2; HR, hormone receptor; PR, progesterone receptor; TNBC, triple-negative breast cancer.

^a^ Race was self-reported. Based on the patient demographics in this study, race was collapsed to three categories for analysis (White, Hispanic, and other).

^b^ Special features included: apocrine, micropapillary, clear cell, mucinous, and metaplastic/squamous.

^c^ Dose dense AC-T: doxorubicin + cyclophosphamide followed or preceded by paclitaxel weekly or every 2 weeks. In one patient treatment changed to CMF after one cycle of AC because of adverse events. In one patient doxorubicin + cyclophosphamide were administered every 3 weeks.

^d^ In one patient treatment changed to CMF after three cycles of docetaxel and cyclophosphamide because of adverse events.

^e^ Incomplete: any dose reduction or change from the planned regimen and/or interruption by >1 week.

^f^ *BRCA1* (n = 4); *BRCA2* (n = 3); *PMS2* deletion (Lynch syndrome), *BARD1*, *NBN*, *PALB2*, *ATM*, *MUYTH* (all, n = 1).

^g^ TCHP: docetaxel-carboplatin-trastuzumab-pertuzumab.

^h^ TCH: docetaxel-carboplatin-trastuzumab.

^i^ Dose dense AC followed by THP: dose dense doxorubicin and cyclophosphamide followed by docetaxel, trastuzumab, and pertuzumab.

### Safety

Nine patients (11.3%) had adverse events possibly attributable to the protocol-mandated tumor biopsy. In a decreasing order of frequency, grade 1 adverse events were bruising (n = 7, 8.8%), breast pain (n = 2, 2.5%), and localized edema (n = 1, 1.3%). Two grade 2 adverse events were reported (biopsy site infection and insomnia; n = 1, 1.3% each). No grade 3 or higher adverse events were reported.

### Outcomes

#### Prespecified Analysis of Outcomes in the Overall Population

##### RFI.

**FIG 2.**
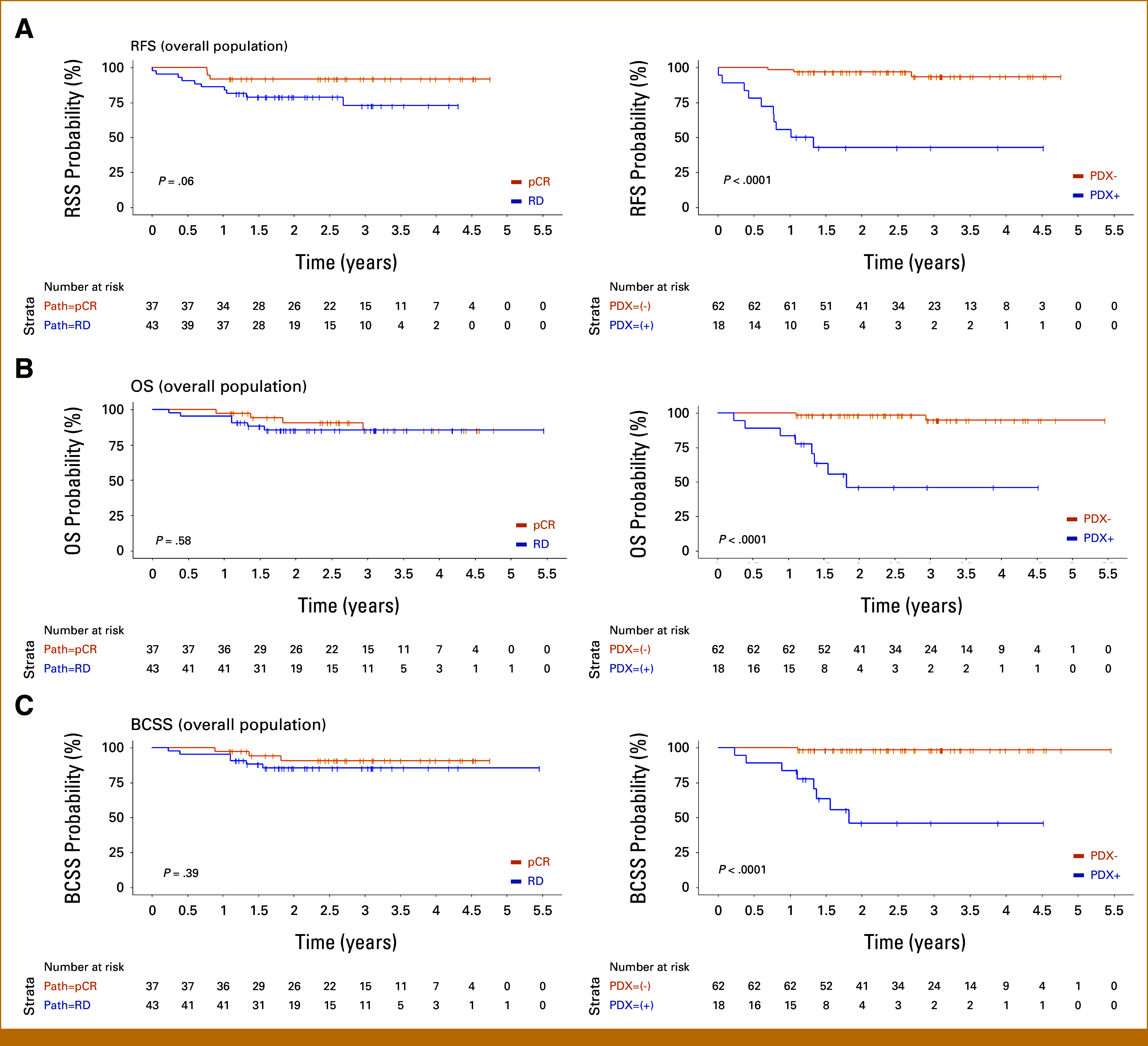
(A) RFS, (B) OS, and (C) BCSS by pCR (left) versus PDX engraftment (right) in the overall population. BCSS, breast cancer-specific survival; OS, overall survival; pCR, pathologic complete response; PDX, patient-derived xenograft; PDX+, successful engraftment; PDX–, no engraftment; RD, residual disease; RFS, relapse-free survival.

With a median follow-up of 2.6 years at the time of data cutoff (range, <1-4.8 years; 2.5 and 2.6 years for patients whose tumors engrafted and did not engraft, respectively), 13 of 80 (16%) patients relapsed, nine of whom relapsed within the first year, and three of whom had achieved a pCR. Achievement of pCR was not significantly associated with relapse in 1 year. By contrast, PDX engraftment was strongly associated with relapse in 1 year: 8 of 18 (44.4%) patients whose tumors engrafted relapsed versus 1 of 62 (1.6%) of patients whose tumors did not engraft (*P* < .0001). Notably, there was successful PDX engraftment from tumors of all three patients who relapsed despite achieving a pCR. All three of these patients relapsed within the first year after definitive surgery and died of metastatic breast cancer within 1 year after their histologic diagnosis of relapse. RFI was statistically significant by PDX status (*P* < .0001), with patients whose tumors engrafted having a hazard ratio (HR) for RFI of 17.5 (95% CI, 4.78 to 64.4; *P* < .001; Fig 2; Table 2).

**TABLE 2.** Summary of Outcomes in the Overall Population

Median Follow-Up at the Time of Data Cutoff	2.6 Years						
RFI	Recurrences (overall)	Recurrences (first year) (95% CI)^a^	Fisher Exact Test (first year)	Median RFI	Log-Rank Test	HR (95% CI)	*P* for HR
All patients	13/80 (16%)	9/80 (11.3%) (5.3 to 20.3)		—			
pCR	3/37 (8.1%)	3/37 (8.1%) (1.7 to 21.9)	NS (*P* = .55)	—	NS (*P* = .06)	3.23 (0.89 to 11.8)	.075
Residual disease	10/43 (23.3%)	6/43 (14%) (5.3 to 27.9)		—			
PDX+	10/18 (55.6%)	8/18 (44.4%) (21.5 to 69.2)	***P* < .0001**	1.18 years	***P* < .0001**	17.5	***P* < .001**
PDX–	3/62 (4.8%)	1/62 (1.6%) (<0.1 to 8.7)		—			

Median Follow-Up at the Time of Data Cutoff	2.6 Years					
OS	Deaths	Fisher Exact Test	Median OS	Log-Rank Test	HR (95% CI)	*P* for HR
All patients	10/80 (12.5%)		—			
pCR	4/37 (10.8%)	Not tested	—	NS (*P* = .58)	1.42 (0.4 to 5.05)	.6
Residual disease	6/43 (14%)		—			
PDX+	8/18 (44.4%)	Not tested	1.82 years	***P* < .0001**	21.1 (4.43 to 101)	***P* < .0001**
PDX–	2/62 (3.2%)		—			

Median Follow-Up at the Time of Data Cutoff	2.6 Years					
BCSS	Deaths	Fisher Exact Test	Median BCSS	Log-Rank Test	HR (95% CI)	*P* for HR
All patients	9/80 (11.3%)		—			
pCR	3/37 (8.1%)	Not tested	—	NS (*P* = .39)	1.83 (0.46 to 7.32)	.4
Residual disease	6/43 (14%)		—			
PDX+	8/18 (44.4%)	Not tested	1.82 years	***P* < .0001**	39.5 (4.91 to 318)	***P* < .0001**
PDX–	1/62 (1.6%)		—			

NOTE. Significant findings are bolded.

Abbreviations: BCSS, breast cancer-specific survival; HR, hazard ratio; NS, not significant; OS, overall survival; pCR, pathologic complete response; PDX, patient-derived xenograft; PDX+, successful engraftment; PDX–, no engraftment; RFI, relapse-free interval.

^a^ Prespecified analysis for recurrence in first year.

##### OS and BCSS.

With a median follow-up of 2.6 years at the time of data cutoff, 10 of 80 patients died, nine of whom died of metastatic breast cancer after recurrence. Eight of the nine breast cancer-related deaths occurred in patients whose tumors engrafted. Overall, 8 of 18 (44.4%) PDX+ patients have died of metastatic breast cancer, compared with only 1 of 62 (1.6%) PDX– patients.

The median OS and BCSS in patients with residual disease and those who achieved pCR has not yet been reached. OS and BCSS by pCR versus residual disease have not reached statistical significance. The median OS and BCSS in PDX+ patients were both 1.82 years (95% CI, 1.37 to not reached); all PDX+ patients who died succumbed to breast cancer. OS and BCSS were statistically significant by PDX engraftment status: the HR for OS and BCSS in PDX+ patients were 21.1 (95% CI, 4.43 to 101; *P* < .001) and 39.5 (95% CI, 4.91 to 318; *P* < .001), respectively.

#### Post Hoc Analysis of Outcomes in the ER-Low HER2-Negative and TNBC Subgroups

**FIG 3.**
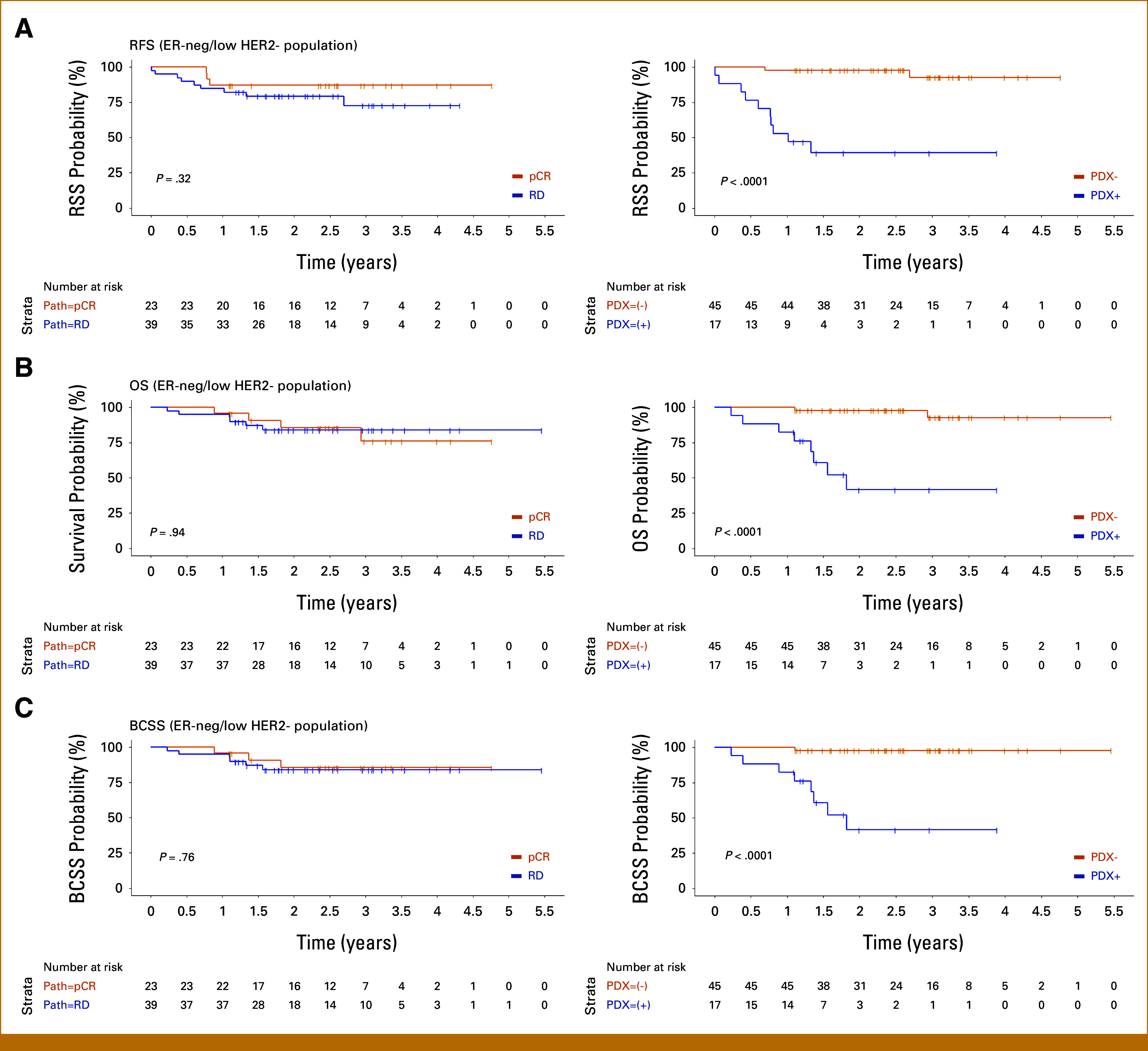
(A) RFS, (B) OS, and (C) BCSS by pCR (left) versus PDX engraftment (right) in the ER-low HER2-negative and TNBC subgroup. BCSS, breast cancer-specific survival; ER, estrogen receptor; HER2, human epidermal growth factor receptor 2; OS, overall survival; pCR, pathologic complete response; PDX, patient-derived xenograft; PDX+, successful engraftment; PDX–, no engraftment; RD, residual disease; RFS, relapse-free survival; TNBC, triple-negative breast cancer.

We next focused on examining outcomes in HER2– patients because exploratory analysis in the small subgroup of HER2(+) patients (n = 18) revealed that neither achievement of pCR nor PDX engraftment were associated with RFI. We note the very small number of relapse events in this subgroup at this time point (n = 1). Unlike TNBC,^8-12^ effective interventions for HER2+ tumors adapted to the presence or absence of residual disease exist,^38^ even if the tumor converts to HER2– after preoperative chemotherapy.^39^ With a median follow-up of 2.9 years at the time of data cutoff (range, 1.05-4.56) in this subgroup, no death events have occurred. Thus, the vast majority of recurrences (12 of 13) and all deaths (n = 10) occurred in patients with ER-low or negative, HER2– breast cancer (n = 62). We therefore examined clinical outcomes in this population, which includes TNBC and ER-low HER2– tumors (Fig 3; Table 3).

**TABLE 3.** Summary of Outcomes in the ER-Low HER2-Negative and TNBC Population

Median Follow-Up at the Time of Data Cutoff	2.6 Years						
RFI	Recurrences (overall)	Recurrences (first year) (95% CI)	Fisher Exact Test (first year)	Median RFI	Log-Rank Test	HR (95% CI)	*P* for HR
All patients	12/62 (19.4%)	9/62 (14.5%) (6.9 to 25.8)		—			
pCR	3/23 (13%)	3/23 (13%) (2.8 to 33.6)	NS (*P* = 1)	—	NS (P = .32)	1.93 (0.42 to 7.13)	.3
Residual disease	9/39 (23%)	6/39 (15.4%) (5.9 to 30.5)		—			
PDX+	10/17 (59%)	8/17 (47%) (23.0 to 72.2)	***P* < .0001**	1.02 years	P < .0001	21.1 (4.54 to 97.7)	***P* < .001**
PDX–	2/45 (4.4%)	1/45 (2.2%) (<0.1 to 11.8)		—			

Median Follow-Up at the Time of Data Cutoff	2.6 Years					
OS	Deaths	Fisher Exact Test	Median OS	Log-Rank Test	HR (95% CI)	*P* for HR
All patients	10/62 (16.1%)		—			
pCR	4/23 (17.4%)	Not tested	—	NS (P = .94)	0.95 (0.27 to 3.37)	>.9
Residual disease	6/39 (15.4%)		—			
PDX+	8/17 (47%)	Not tested	1.82 years	P < .0001	17.6 (3.66 to 84.8)	***P* < .001**
PDX–	2/45 (4.4%)		—			

Median Follow-Up at the Time of Data Cutoff	2.6 Years					
BCSS	Deaths	Fisher Exact Test	Median BCSS	Log-Rank Test	HR (95% CI)	*P* for HR
All patients	9/62 (14.5%)		—			
pCR	3/23 (13%)	Not tested	—	NS (P = .76)	1.24 (0.31 to 4.98)	.8
Residual disease	6/39 (15.4%)		—			
PDX+	8/17 (47%)	Not tested	1.82 years	P < .0001	31.9 (3.95 to 257)	***P* < .001**
PDX–	1/45 (2.2%)		—			

NOTE. Significant findings are bolded.

Abbreviations: BCSS, breast cancer-specific survival; ER, estrogen receptor; HER2, human epidermal growth factor receptor 2; HR, hazard ratio; NS, not significant; OS, overall survival; pCR, pathologic complete response; PDX, patient-derived xenograft; PDX+, successful engraftment; PDX–, no engraftment; RFI, relapse-free interval; TNBC, triple-negative breast cancer.

##### RFI.

Achievement of pCR versus residual disease was not significantly associated with 1-year relapse; however, 8 of 17 (47%) PDX+ patients relapsed within 1 year after definitive surgery compared with 1 of 45 (2.2%) of PDX– patients (*P* < .0001). RFI was statistically significant by PDX engraftment status (*P* < .0001), with PDX+ patients having a HR for RFI event of 21.1 (95% CI, 4.54 to 97.7).

##### OS and BCSS.

With a median follow-up of 2.5 years at data cutoff (range, 0.23-5.46 years) for OS and BCSS*,* 10 of 62 (16.1%) patients died, nine of whom died of metastatic breast cancer. Eight of nine breast cancer–related deaths occurred in PDX+ patients. Overall, 8 of 17 (47%) of ER-low or negative, HER2– patients whose tumors were PDX+ died of breast cancer, whereas only 1 of 45 (2.2%) of PDX– patients have died of breast cancer.

The median OS and BCSS in patients with residual disease and those who achieved pCR has not yet been reached. OS and BCSS by pCR versus residual disease have not reached statistical significance. The median OS and BCSS in PDX+ patients were both 1.82 years (95% CI, 1.33 to not reached), and all PDX+ patients who died succumbed to breast cancer. OS and BCSS were statistically significant by PDX status, and the HR for OS and BCSS in PDX+ ER-low or negative, HER2– patients were 17.6 (95% CI, 3.66 to 84.8; *P* < .001) and 31.9 (95% CI, 3.95 to 257; *P* < .001), respectively.

#### Outcomes of Patients Who Relapsed

**FIG 4.**
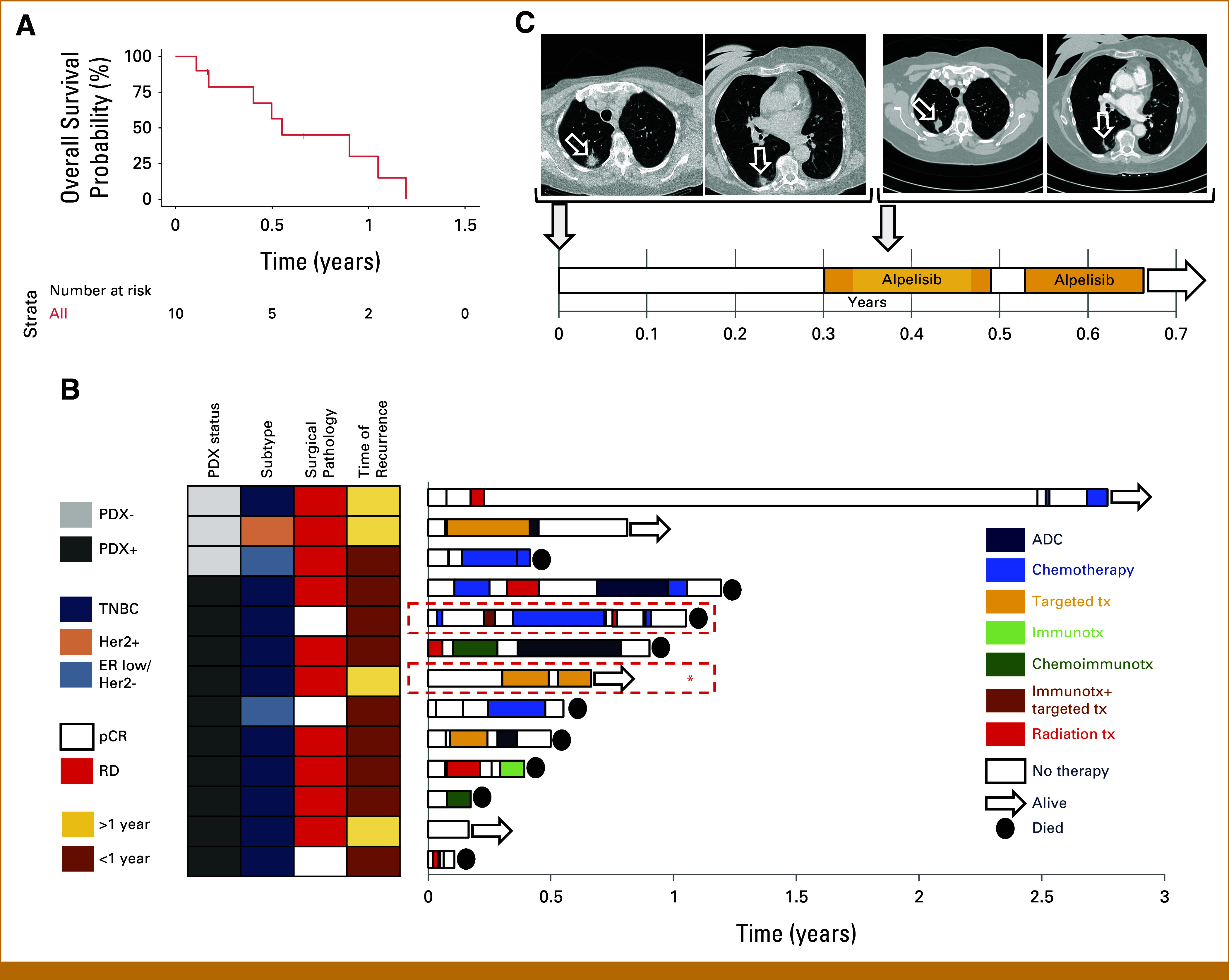
(A) Overall survival time of the PDX+ patients who relapsed, measured from the time of histologic diagnosis of relapse. (B) Overview of the characteristics and treatment timelines of tumors that relapsed, stratified by PDX engraftment. Time of recurrence was the interval between definitive surgery and histologic diagnosis of recurrence. Patients were treated per physician discretion. The two PDX+ patients for whom PDX-derived organoid-based drug profiling was performed on recurrence for informed treatment selection are highlighted in red boxes on the right. The first patient has been discussed elsewhere^17^; the second patient (marked with ^a^) is shown in detail in (C). (C) Clinical course of a patient with PDX+ metaplastic breast cancer after development of recurrent (metastatic) disease (day 0) where treatment was informed by prospective genomic and PDX-derived organoid-based drug profiling studies. ER, estrogen receptor; HER2, human epidermal growth factor receptor 2; pCR, pathologic complete response; PDX, patient-derived xenograft; PDX+, successful engraftment; PDX–, no engraftment; RD, residual disease; TNBC, triple-negative breast cancer.

This study so far has only captured early relapses. PDX+ patients who had recurrence (n = 10) relapsed early, with 80% relapsing within the first year following definitive surgery. These patients had an exceptionally unfavorable prognosis: the median survival following the histologic diagnosis of recurrence was only 0.55 years (95% CI, 0.4 to not reached; Figs 4A and 4B).

Three PDX– patients have had a relapse so far. One patient relapsed 2.7 years after her definitive surgery but is still alive 2.76 years after histologic diagnosis of recurrence. A second patient relapsed 1.05 years after her definitive surgery; at data cutoff, she was still alive without having received anticancer therapy for the preceding 6 months. A third PDX– patient relapsed 0.7 years after her definitive surgery, and her survival after the diagnosis of recurrence was 5 months.

In two PDX+ patients who relapsed, we leveraged our PDX-derived organoid-based functional precision oncology platform^17^ to inform treatment selection. The first case has been discussed elsewhere.^17^ In the second case (a patient with metastatic metaplastic breast cancer), genomic profiling of the residual tumor revealed a *PIK3CA* H1047R mutation and drug profiling predicted a response to alpelisib; multiple other targeted agents including everolimus, and chemotherapies did not appear to be effective. The patient received alpelisib as monotherapy. Despite dose reductions, interruptions, and modifications, her disease remains in remission for 4.4+ months (Fig 4C). Because PDX+ patients relapsed early and had an exceptionally aggressive disease, we were unable to perform drug screening fast enough to inform treatment selection in other patients. A separate ongoing clinical trial has been launched to address this limitation, where drug screening is performed before recurrence (*TOWARDS-II*; ClinicalTrials.gov identifier: NCT05464082).

### Univariate Analyses

Contrary to our hypothesis, achievement of pCR and PDX engraftment were not statistically correlated with each other, in either the overall population (prespecified; odds ratio [OR], 1.46 [95% CI, 0.45 to 5.09) or in the ER-low or negative, HER2– subgroup (post hoc; OR, 1.11 [95% CI, 0.31 to 4.37]). Patients with residual cancer burden (RCB) 3 (minimal treatment effect) were more likely to have had tumors that engrafted in mice (50% *v* 19%, 31%, and 16% for RCB 0, 1, and 2, respectively); however, the relationship between PDX engraftment and RCB class did not reach statistical significance (Fisher exact test; *P* = .3).

By univariate analysis in both the overall population and the ER-low or negative, HER2– subgroup, PDX engraftment and RCB were associated with relapse, death of any cause, and death from breast cancer, with PDX engraftment best discriminating the patients at highest risk of an event (Appendix Table A1).

We conducted post hoc analysis to determine the relative accuracy of PDX engraftment or pCR in predicting early recurrence in the overall population. Recurrence was predicted to occur from PDX+ tumors or when pathology showed residual disease. No recurrence was predicted from PDX– tumors or with pCR. PDX engraftment correctly predicted 1-year recurrence in 69 of 80 (86%) patients versus pCR, where residual disease correctly predicted 1-year recurrence in 40 of 80 (50%) patients. The *P* value for a difference in overall accuracy is *P* = 3.6e-06 (Wilcoxon signed rank test). Correct predictions for PDX engraftment and pCR were statistically independent (*P* = 1.0, Fisher exact test). We found no significant difference in the sensitivity of prediction between PDX engraftment and pCR (perhaps because of only nine recurrences at 1 year), but found a highly significant difference in specificity of prediction. PDX engraftment accurately predicted no 1-year recurrence in 61 of 71 (86%) patients versus pCR, which accurately predicted no 1-year recurrence in only 34 of 71 (48%) patients (*P* = 5.2e-06; Wilcoxon signed rank test). The contingency tables for these analyses are in Appendix Tables A2-A4. After taking into consideration the PDX engraftment status, only RCB class added to the prediction of an event (Appendix Table A5). The number of events, however, is currently too small to include many predictors in a multivariate analysis.

## DISCUSSION

We report that in a prospective, blinded, noninterventional study of 80 newly diagnosed, nonmetastatic patients with ER-negative or low breast cancer, PDX engraftment is strongly prognostic for early recurrence and death from breast cancer. In this study, we found PDX engraftment was a more specific indicator of recurrence 1 year after completion of neoadjuvant therapy and surgery than pCR. The HR for OS and BCSS in PDX+ patients in the overall population was 21.1 and 39.5, respectively. In the TNBC or ER-low, HER2– population, the HR was 17.6 for OS and 31.9 for BCSS. So far, we do not detect a correlation between PDX engraftment and clinical outcomes in the HER2+ population. Although the follow-up for relapse and survival is still short at this time, the study revealed that PDX engraftment can, at least, capture early metastatic relapses that carry an exceptionally dismal prognosis. All living patients continue to be followed to determine whether PDX engraftment is also prognostic for later recurrences, and the analysis of the 3-year end points will be reported in a future publication.

Our results differ from a previous publication that reported no correlation between PDX engraftment and breast cancer outcomes (BEAUTY).^40^ Many differences exist between the BEAUTY and TOWARDS studies. First, the patient population in BEAUTY included >10% ER-positive cases, which were excluded in the TOWARDS study. Second, three key methods for PDX engraftment differed. In TOWARDS, we surgically implanted tumor fragments into cleared inguinal mammary fat pads of NOD/SCID mice and did not give hormone supplements. In BEAUTY, tumor fragments were injected subcutaneously with a trochar into the flanks of mice that were treated with estrogen, and the mice used were a mixture of NOD/SCID and NSG strains. The take rate of breast cancer PDX engraftment is higher in NSG mice than in NOD/SCID mice^21^ because of lack of natural killer cells in the NSG strain.^41^ Indeed, the BEAUTY authors reported significantly higher PDX engraftment in NSG versus NOD/SCID mice.^40,42^ The overall take rate reported for TNBC in BEAUTY (all mice) was 51.3%; the take rate of TNBC or ER-low HER2– tumors in TOWARDS (NOD/SCID mice) was 27.4% (17/62). Thus, it is possible that orthotopic implantation of tumors into NOD/SCID mice without hormone supplementation provides more stringent engraftment conditions that can readily discriminate the most aggressive tumors.

Our study suggests that PDX engraftment can prospectively identify the most aggressive TNBC or ER-low, HER2– tumors and that these patients could be prioritized for additional therapy to prevent recurrence. The models generated in this study may also be leveraged to optimize adjuvant treatment by sparing additional therapies in patients not likely to recur and by selecting therapies that are more effective at eliminating disease in patients with substantially higher risk for relapse. Indeed, the models generated from these aggressive breast tumors are being used for drug screening to identify more effective therapies in this patient population.^17^

A limitation of our study is that the treatment paradigm in early stage TNBC has changed with the incorporation of immunotherapies. TOWARDS preceded the approval of pembrolizumab in the neoadjuvant and adjuvant setting in nonmetastatic TNBC,^5,6^ thus no patients received pembrolizumab. Although tumors grown as PDXs reproduce the tumor microenvironment better than cell culture,^43^ interactions with human immune cells are lacking. Attempts, including our own,^44^ to humanize the immune system in PDX-bearing mice are ongoing, but are not yet tractable to predict effects of immunotherapy.^45^ Despite this, the advent of immunotherapies has not eliminated breast cancer recurrence and, in practice, the incremental benefits in efficacy may be compromised by high incompletion rates because of toxicities. Any differences in the prognostic application of PDX engraftment, now that immunotherapy is SOC, will be captured in our ongoing follow up trial, TOWARDS-II (ClinicalTrials.gov identifier: NCT05464082).

Another limitation of our findings, despite the large and significant effect size, is that PDX engraftment is impractical as a standard prognostic test. A definitive and specific biomarker of PDX engraftment has not yet been discovered, but is an area of investigation. Future work in this area will be key to translate this finding to a feasible assay that can be conducted for all ER-negative/low, HER2– patients. Finally, identification of the most aggressive breast cancers is most impactful if there are effective treatments for these tumors. Use of PDX and other models representing these aggressive tumors to identify new, more effective drugs is urgently needed.

## APPENDIX

**TABLE A1.** Univariate Screen to Detect Predictors of Relapse, Death of Any Cause, and Death of Breast Cancer

Predictor Variable	Outcome Variable		
	RFS	OS	BCSS
Overall population			
pCR *v* residual disease	0.053	0.583	0.381
PDX engraftment	**1.41 × 10** ^**–**^ **^6^**	**9.09 × 10** ^**–**^ **^6^**	**2.33 × 10** ^**–**^ **^6^**
RCB class	**0.008**	**0.006**	**0.007**
Race (White, Hispanic, other)	**0.049**	0.103	0.141
Age >53 years	0.392	0.482	0.736
Clinical stage on diagnosis (I, II ,III)	**0.02**	0.124	0.096
Nodal status (positive or negative)	0.414	0.304	0.528
HR status (HR-low or negative)	0.528	0.833	0.984
HER2 status (±)	0.087	**0.014**	**0.021**
Grade (2 or 3)	0.94	0.591	0.647
Size of largest lesion at diagnosis	0.213	0.468	0.419
Preoperative treatment complete (yes/no)	0.768	0.913	0.913
HR-low or negative/HER2– subgroup			
pCR *v* residual disease	0.3	0.938	0.381
PDX engraftment	**2.22 × 10** ^**–**^ **^6^**	**3.31 × 10** ^**–**^ **^5^**	**2.33 × 10** ^**–**^ **^6^**
RCB class	**0.015**	**0.01**	**0.007**
Race (White, Hispanic, other)	**0.049**	0.082	0.141
Age >53 years	0.672	0.609	0.736
Clinical stage on diagnosis (I, II, III)	0.055	0.105	0.096
Nodal status (positive or negative)	0.595	0.28	0.528
HR status (HR-low or negative)	0.996	0.776	0.984
Grade (2, 3)	0.951	0.581	0.647
Size of largest lesion at diagnosis	0.181	0.216	0.419
Preoperative treatment complete (yes/no)	0.552	0.923	0.913

NOTE. Cox likelihood ratio test *P* values for statistical significance as a predictor for an event in the overall population and HR-low or negative/HER2-negative subgroup. *P* < .05 highlighted in bold.

Abbreviations: BCSS, breast cancer-specific survival; HER2, human epidermal growth factor receptor 2; HR, hormone receptor; OS, overall survival; pCR, pathologic complete response; PDX, patient-derived xenograft; RCB, residual cancer burden; RFS, relapse-free survival; TNBC, triple-negative breast cancer.

**TABLE A2.** Contingency Table for Analysis of Accuracy of PDX Engraftment and pCR in Assessing 1-Year Recurrence

PDX Accurate at Predicting 1-Year Recurrence?	pCR Accurate at Predicting 1-Year Recurrence?		Total
	No	Yes	
No	6	5	11
Yes	34	35	69
Total	40	40	80

Abbreviations: pCR, pathologic complete response; PDX, patient-derived xenograft.

**TABLE A3.** Contingency Table for Analysis of Sensitivity of PDX Engraftment and pCR to Predict 1-Year Recurrence for Those That Recurred

Is PDX Correct at Predicting 1-Year Recurrence for Those That Recurred?	Is pCR Correct at Predicting 1-Year Recurrence for Those That Recurred?		Total
	No	Yes	
No	0	1	1
Yes	3	5	8
Total	3	6	9

Abbreviations: pCR, pathologic complete response; PDX, patient-derived xenograft.

**TABLE A4.** Contingency Table for Analysis of Specificity of PDX Engraftment and pCR to Predict No 1-Year Recurrence for Those That Did Not Recur

Is PDX Correct at Predicting No 1-Year Recurrence, for Those That Did Not Recur?	Is pCR Correct at Predicting No 1-Year Recurrence for Those That Did Not Recur?		Total
	No	Yes	
No	6	4	10
Yes	31	30	61
Total	37	34	71

Abbreviations: pCR, pathologic complete response; PDX, patient-derived xenograft.

**TABLE A5.** Effect of Adding One Additional Variable to PDX Engraftment Multivariate Analysis (restricted to predictors with *P* < .1 for at least one of the univariate analyses)

Predictor Variable	Outcome Variable		
	RFS	OS	BCSS
Overall population			
pCR *v* residual disease	0.081	0.832	0.55
RCB class	**0.012**	**0.015**	**0.003**
Clinical stage on diagnosis (I, II, III)	0.141	0.43	0.38
HR status (HR-low or negative)	0.3	0.682	0.84
HER2 status (±)	0.437	0.07	0.139
HR-low or negative/HER2(–) subgroup			
pCR *v* residual disease	0.309	0.742	0.55
RCB class	**0.009**	**0.03**	**0.003**
Race (White, Hispanic, other)	0.071	0.124	0.269
Clinical stage on diagnosis (I, II, III)	0.319	0.481	0.38

NOTE. *P* < .05 highlighted in bold.

Abbreviations: BCSS, breast cancer-specific survival; HER2, human epidermal growth factor receptor 2; HR, hormone receptor; OS, overall survival; pCR, pathologic complete response; PDX, patient-derived xenograft; RCB, residual cancer burden; RFS, relapse-free survival.
